# Inequities in Childhood Vaccination Coverage in Zhejiang, Province: Evidence from a Decomposition Analysis on Two-Round Surveys

**DOI:** 10.3390/ijerph15092000

**Published:** 2018-09-13

**Authors:** Yu Hu, Hui Liang, Ying Wang, Yaping Chen

**Affiliations:** Institute of Immunization and Prevention, Zhejiang Center for Disease Control and Prevention, Hangzhou 310000, China; hliang@cdc.zj.cn (H.L.); ywang@cdc.zj.cn (Y.W.)

**Keywords:** vaccination, coverage, inequality, concentration index, decomposition

## Abstract

*Objective*: The objectives of this study were to determine the degree and risk factors of the inequity in the childhood coverage of full primary immunization (FPI) in Zhejiang province. *Method*: We used data from two rounds of vaccination coverage surveys among children aged 24–35 months conducted in 2014 and 2017, respectively. The household income per month was used as an index of socioeconomic status for the inequality analysis. The concentration index (CI) was used to quantify the degree of inequality, and the decomposition approach was applied to quantify the contributions from demographic factors to inequality in the coverage of FPI. *Results*: The coverage rates of FPI were 80.6%, with a CI value of 0.12028 for the 2014 survey, while the coverage rates of FPI were 85.2%, with a CI value of 0.10129 for the 2017 survey. The results of decomposition analysis suggested that 68.2% and 67.1% of the socioeconomic inequality in the coverage of FPI could be explained by the mother’s education level for the 2014 and 2017 survey, respectively. Other risk factors including birth order, ethnic group, mother’s age, maternal employment status, residence, immigration status, GDP per-capita, and the percentage of the total health spending allocated to public health could also explain this inequality. *Conclusion*: The socioeconomic inequity in the coverage of FPI still remained, although this gap was reduced between 2014 and 2017. Policy recommendations for health interventions on reducing the inequality in the coverage of FPI should be focused on eliminating poverty and women’s illiteracy.

## 1. Introduction

The United Nations Sustainable Development Goals include a focus on the improvement of children’s health and survival as a global priority [1]. Such goals have led to an increase of investments aiming to promote the accessibility and affordability of childhood health intervention programs, particularly regarding vaccination services and nutrition. The World Health Organization (WHO) launched the Expanded Program on Immunization (EPI) in 1974 as a public health initiative targeting the improvement of childhood health [2]. The Chinese EPI was started in 1978 with 4 vaccines, and today totals 11 vaccines. The primary immunization schedule includes one dose of Bacillus Calmette–Guérin vaccine (BCG) scheduled at birth; three doses of hepatitis B vaccine (HBV) scheduled at birth, 1 month, and 6 months of age; three doses of diphtheria-tetanus-pertussis combined vaccine (DTP) scheduled at 3, 4, and 5 months of age; three doses of polio vaccine (PV) scheduled at 2, 3, and 4 months of age; one dose of measles-containing vaccine (MCV) scheduled at 8 months of age; and one dose of Japanese encephalitis vaccine (JEV) scheduled at 8 months of age. It is recommended that a child receive the primary required immunizations before 12 months of age. All vaccines of the Chinese EPI are provided free of charge [3]. The remarkable achievement of the Chinese EPI is the significant reduction in incidences of vaccine-preventable diseases (VPDs) among children. For example, the rate of positive hepatitis B surface antigen test results among children under 5 years of age reduced from 4.05% in 1992 to 0.78% in 2014. In 2008, the Chinese advisory committee on immunization practice (CACIP) set the coverage goal of 90% for all antigens, which is required to reach herd immunity and stop the transmission of VPDs. 

The distribution of health inequity has become more prominent, as the average indicator is inadequate in reflecting the performance of public health interventions. Traditionally, studies examining the inequities in health outcomes applied the concentration index (CI) and concentration graph approaches [4,5,6]. Additionally, some of those studies decomposed the general inequity to detect the pathways of the observed childhood health inequities [7,8,9,10]. In the 2011 provincial coverage survey, we found that each antigen reached the goal of 90% at 12 months old. However, a full immunization coverage of 69.3% was found in Yiwu city among a similar target population in 2011. As such, a potential inequity in vaccination coverage within Zhejiang province may exist, and could have a substantially negative influence on the EPI. For policy-making purposes, it is critical to detect and understand the risk factors which contribute to the EPI implementation and its effectiveness in the sub-province. Achieving universal coverage of childhood immunization involves creating equal accessibility for all eligible children, in addition to identifying the vulnerable children who are at risk of under-immunization. To our knowledge, some socioeconomic or demographic factors were determined to influence the vaccination coverage in previous studies, and most of them were at child-specific [11,12], parental [13,14], and household levels [15,16]. However, there were few published papers analyzing the inequity of the vaccination coverage and its related risk factors in Zhejiang province. 

In this study, we employed data from the 2014 and 2017 vaccination coverage surveys of Zhejiang province to evaluate the socioeconomic inequity in primary immunization among children aged 24–35 months. We then decomposed the inequity by quantifying the contribution attributable to demographic variables.

## 2. Materials and Methods

### 2.1. Study Area

Zhejiang is a middle-income, developing province on the east coast of China, with an area of 1105.5 square kilometers and a population of approximately 70 million. Administratively, it is divided into 11 cities, 90 counties, and 1319 towns. The average annual birth rate was around 1.1% in the last five years, with an estimated 558,520 births in 2012 and 639,652 births in 2015, respectively. The increase in annual birth was mainly due to the opening of the two-child policy in China. 

### 2.2. Survey Design and Sampling 

This study used the two rounds of Zhejiang provincial vaccination coverage surveys of children 24–35 months old in 2014 (born between 1 September 2011 and 31 August 2012) and 2017 (born between 1 September 2014 and 31 August 2015, respectively). 

Both surveys applied the household-based cluster survey method recommended by the WHO [17]. The sample size of the two surveys was calculated based on the formula as follows:

$$N=deff\times \frac{z_{\left(1-\frac{\alpha }{2}\right)}^{2}\times p\times \left(1-p\right)}{d^{2}}.$$

The parameters were set as a two-tailed α error of 5%, a permissible error (d) of 0.08, a design effect (deff) of 2, and expected coverage of full primary immunization (FPI) at 0.7 for the 2014 survey and 0.9 for the 2017 survey, respectively. As such, the final sample size required for each city was 252 (2014 survey) or 70 (2017 survey) eligible children, corresponding to 2772 (2014 survey) or 770 (2017 survey) children at the provincial level, respectively.

### 2.3. Field Survey Procedures

In the 2014 coverage survey, the sample size was divided into six towns of 42. The detailed procedures included four steps: First, six towns for each city were selected from a list of all towns (with the population size), on the basis of the probability proportional to population size. Second, one community was randomly selected from each town through a simple ballot from the list of all communities. Third, the index household was selected randomly from the list of all households by using a table of random numbers. Fourth, the subsequent households were selected by turning to the right while exiting the index household and visiting the adjacent one. Only one eligible child per household was chosen. Households were re-visited if there was they were occupied but there was no response upon initial visit. The closest community was selected to survey the rest of the children if an adequate sample could not be obtained in the selected community. In the 2017 coverage survey, the sample size was divided into seven towns of 10. The detailed procedures were similar to those in the 2014 coverage survey.

### 2.4. Data Collection 

Vaccination data were transcribed from the immunization cards, and were validated through the Zhejiang provincial immunization information system (ZJIIS). The main function of ZJIIS can be found in our previous report [18]. Demographic and socioeconomic information were collected through a pre-tested standard questionnaire developed by the Zhejiang provincial center for disease control and prevention (ZJCDC). Specifically, the demographic variables included four aspects: child-level (gender, birth place, birth order, and ethnic group), mother-level (education level, age, and employment status), household-level (residence and socioeconomic status), and city-level (gross domestic product (GDP) per-capita, and the percentage of the total health spending allocated to public health).

### 2.5. Definitions

The FPI was defined as a child in receipt of the 12 scheduled vaccine doses before 12 months of age, and it was also defined as a dependent and binary variable—namely, whether a child received all 12 vaccine doses by 12 months of age or not (fully immunized = 1, not fully immunized = 0). The socioeconomic distribution was categorized into tertiles according to the household income per month, with the first tertile representing the poorest group and the last tertile representing the least-poor group.

### 2.6. Analytical Method

We used STATA MP version 14.0 (StataCorp, 2015, Stata statistical software, College Station, TX, USA) for data analysis. The procedures of data analysis included three steps: First, we described the distribution of the demographic variables, the socioeconomic characteristic, the FPI coverage, as well as the coverage of individual doses. Second, the socioeconomic inequity of the coverage of FPI was evaluated through the CI and the concentration curve (CC). The cumulative FPI coverage (*y*-axis) was plotted against the cumulative percentage of the population, which was ranked by household income per month, beginning with the poorest and ending with the least poor (*x*-axis) [19]. If there was no inequity in the FPI coverage, the CC would be a 45° line running from the bottom left-hand corner to the top right-hand corner. It was known as the equity line or the diagonal line. If the higher/lower FPI coverage favored the poorer, the CC would lie above/below the diagonal line. The further the CC deviated from the diagonal line, the greater the degree of inequity. The CI was defined as twice the area between the concentration curve and the diagonal line. The range of CI was from −1 to +1, which occurred if the FPI coverage was concentrated in the least poor group or the poorest group, respectively. The CI was calculated by the following formula: $\mathrm{CI}=\frac{2}{\mu }\mathrm{cov}\left(h,r\right)$, where *h* and *μ* are the FPI coverage and its mean, and $r=\frac{i}{N}$ is the rank of the socioeconomic status, with *i* = 1 for the greatest disadvantage and *i* = *N* for the least. Third, we applied the logistic regression model to detect the significant risk factors associated with the FPI coverage, including all of the significant variables in the univariate analysis (*χ*^2^ test) and using a *p* value of <0.1 as a screening criterion. The logistic regression analysis was performed based on the formula $\mathrm{Log}\left({\hat{p}}_{i}\right)=\sum_{k}\beta_{k}x_{ki}+\varepsilon_{i}$. The $\mathrm{Log}\left({\hat{p}}_{i}\right)$ is the logistic of the predicted probability of being FPI, $\beta_{k}$ is the coefficient for the risk factor $x_{k}$, and $\varepsilon_{i}$ is the residual estimate. As such, the CI could be re-written as $\mathrm{CI}=\sum_{k}\left(m_{k}{\bar{x}}_{k}/\mu \right)CI_{k}+GC_{\varepsilon }/\mu$, where μ is the mean of the FPI coverage, ${\bar{x}}_{k}$ is the mean of the $x_{k}$, and $CI_{k}$ is the CI of the $x_{k}$. The marginal effect is defined as $m_{k}=\lambda \left(\sum_{k}\beta_{k}{\bar{x}}_{k}\right)\beta_{k}$. λ() is the logistic density function. $m_{k}$ means the average change in the probability of the FPI coverage when the risk factor $x_{k}$ changed by one unit. $GC_{\varepsilon }$ means the CI of the residual ($\varepsilon_{i}$), which was calculated as follows: $GC_{\varepsilon }=\frac{2}{N}\overset{N}{\underset{i=1}{\sum }}\varepsilon_{i}r_{i}$. $\left(m_{k}{\bar{x}}_{k}/\mu \right)CI_{k}$ is the contribution of the CI. We applied a “bootstrap” procedure in a five-step manner to obtain the standard errors (S.E.s) of the CI [20].

### 2.7. Ethical Considerations

The two rounds of Zhejiang provincial vaccination coverage surveys were approved by the ethical review board of Zhejiang provincial CDC (T-043-R for the 2014 survey and T-060-R for the 2017 survey). Written informed consent was obtained from a parent or a legal caregiver of each eligible child enrolled in this study.

## 3. Results

In total, 2772 children and 770 children aged 24–35 months participated in the 2014 and 2017 vaccination coverage surveys of Zhejiang province, respectively. Table 1 presents the demographic and socioeconomic characteristics of the participants. Of the children included in the 2014 coverage survey, 50.3% were male, 70.4% were first-born, 91.1% were of Han ethnicity, and 40.1% were migrant. Most of the surveyed mothers were <30 years of age (65.9%), had at least secondary level education (61.0%), and had a fixed job (86.8%). Over half of the surveyed households were located in rural areas (50.9%). Of the children included in the 2017 coverage survey, 50.5% were male, 65.2% were first-born, 91.9% were of Han ethnicity, and 37.7% were migrant. Most of the surveyed mothers were <30 years of age (88.6%), had at least secondary level education (59.3%), and had a fixed job (79.7%). Over half of the surveyed households were located in urban areas (50.4%).

The coverage rates of FPI at the provincial level were 80.6% and 85.2% for the 2014 survey and the 2017 survey, respectively. In the univariate analysis, the risk factors of the coverage of FPI included the birth order of child, the ethnic group of child, the maternal education level, the maternal employment status, the household residence, the immigration status, and the socioeconomic status for both surveys. Furthermore, the mother’s age was found as a significant determinant of the coverage of FPI in the 2017 survey only. 

**Figure 1:**
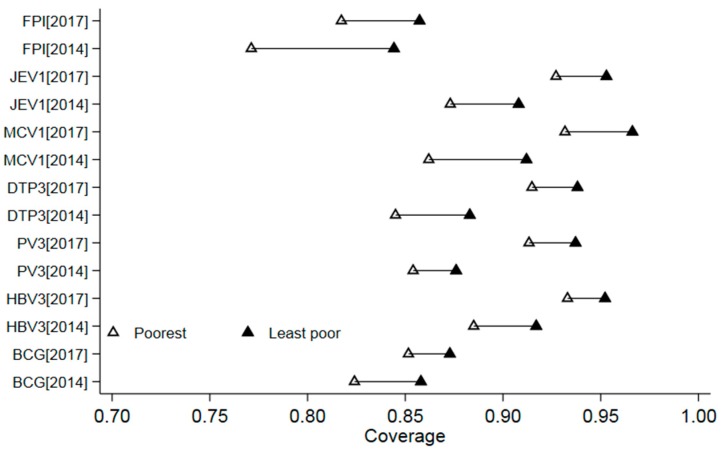
The distribution of coverage of the individual vaccine and FPI by socioeconomic status in the 2014 and 2017 coverage surveys (Notes: HBV3 means the 3rd dose of the hepatitis B vaccine (HBV); PV3 means the 3rd dose of the polio vaccine (PV); DTP3 means the 3rd dose of the diphtheria-tetanus-pertussis combined vaccine (DTP); MCV1 means the 1st dose of the measles-containing vaccine (MCV); JEV1 means the 1st dose of the Japanese encephalitis vaccine (JEV)).

Figure 1 shows the disparities in the coverage of individual vaccine doses and FPI in the two surveys. In general, the coverage for the individual and the FPI was consistently lower in the poorest group than the least poor group for both the 2014 and the 2017 survey. The largest differences were found in the coverage of FPI, with 7.3% for the 2014 survey and 4.0% for the 2017 survey, respectively. 

**Figure 2:**
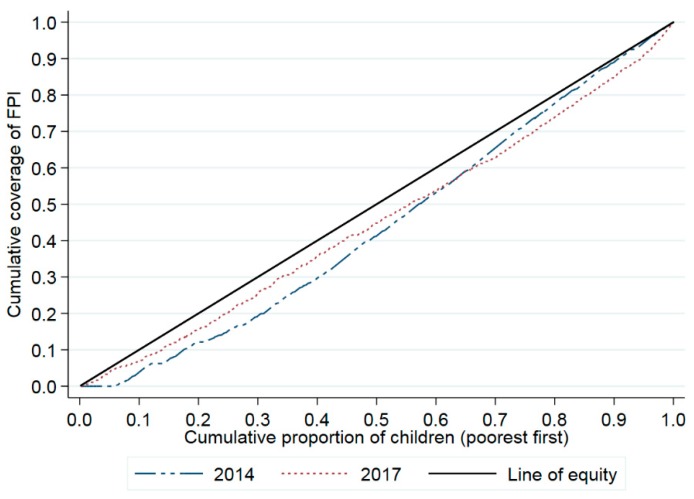
The concentration curve of inequality in the coverage of FPI in the 2014 and 2017 coverage surveys.

The CI values were 0.12028 (95% CI: 0.10852–0.13175) for the 2014 survey and 0.10129 (95% CI: 0.095914–0.12068) for the 2017 survey, which suggests that the coverage of FPI significantly favored children with a relatively higher socioeconomic status (Figure 2). 

Table 2 and Table 3 present the CIs of each determinant and the contributions to the inequality in the coverage of FPI. For both the 2014 and the 2017 surveys, the risk factors that increased the concentration of the coverage of FPI in the least poor group included birth order, Han ethnic group, higher maternal education level, stay-at-home mothers, living in rural areas, resident children, high GDP per-capita, and high percentage of the total health spending allocated to public health. Additionally, the “mothers under 30 years of age” category was also found to be a significant risk factor for increasing the concentration of the coverage of FPI in the least poor group. 

## 4. Discussion

Although the EPI is considered as one of the most cost-effective strategies in public health, as it has substantially reduced incidences of VPDs worldwide, many developing areas including China still face the challenge of low vaccination coverage in the sub-population or sub-geopolitical area, which is an obvious risk factor of the endemicity of VPDs [21,22]. By analyzing the data from two rounds of coverage surveys in 2014 and 2017, we found an improvement in the coverage of FPI among children aged 24–35 months, with an increase of 5%. This might be explained by the policy or priority changes in the vaccination service in Zhejiang province in recent years. First, the financial incentives to vaccination providers with 5 CNY per dose encouraged the providers to actively search drop-out or under-immunized children. Second, a training program toward all providers in Zhejiang province was conducted during 2014–2015. Third, over 90% of the vaccination providers extended their working time or increased the frequency of sessions to improve the accessibility of the vaccination service since 2014.

Large disparities in the coverage of FPI were observed in both of the surveys, and the inequity in the coverage of FPI decreased between 2014 and 2017. The inequity gap closing between these two surveys might be due to the improvements from the providers, as mentioned above, or from the recipients. However, due to data limitations and the study design, there was no direct evidence to prove which intention contributed to the improvement in the inequity in recent years. The results also revealed that low socioeconomic status had a significant negative effect on the probability of FPI. Though the childhood vaccination service was free (which should theoretically narrow the inequality related to socioeconomic status), parents still faced some challenges in having their children vaccinated. First, mothers might face a significant indirect cost (travel fee or working time lost) to access the vaccination clinic. Second, socioeconomically disadvantaged people prefer to work to improve their living standards, which would increase the demand for caregivers’ time in income-generating activities instead of completing the vaccination schedule for their children [16,23]. 

After the decomposition analysis of the risk factors of inequity in the coverage of FPI, a mother’s education level stood out with the largest contribution of 38% or 39% for the 2014 survey and the 2017 survey, respectively. Improving a mother’s education level has been promoted worldwide as a mechanism for enhancing the outcome of child health, especially in developing countries [24,25]. In fact, a growing number of published studies reveal the significant effects that an increased level of maternal education has on improving childhood health outcomes. The results from the decomposition analysis were consistent with the findings of those previous reports [26,27,28], which indicated that an additional year of a mother’s education exerted a positive effect on the probability of a child receiving the primary vaccination. The educational attainment of a mother enhanced the accessibility of information on vaccinations, and facilitated the communication between mothers and health providers, leading to a better understanding of vaccination schedules and practice. Our findings suggest that it is necessary to establish a system whereby mothers are informed about the importance of vaccinations, in addition to relying on the traditional education system. Specifically, creating vaccination awareness among mothers or other household decision-makers must become a core strategy for improving coverage, irrespective of literacy status.

Our results showed the presence of significant rural–urban disparity in the probability of the FPI in both survey rounds. Specifically, children living in rural areas were more likely to complete the primary immunization schedule. Our results differed from the previous reports from developing countries [12,13], where there was a significant rural disadvantage in child immunization coverage. However, other studies found substantial barriers to vaccination coverage in urban settings in spite of the physical access to health facilities in urban areas [29,30]. The difference in the public health delivery system between rural and urban areas might contribute to the urban immunization disadvantage in Zhejiang province. Over the last three decades, a rapid pace of urbanization occurred due to the high-speed economic development in Zhejiang province. Most doctors working in urban vaccination clinics did not have time to explain the importance of vaccination to parents. The direct connection between the parents and providers was lacking in urban areas, and the doctors were less likely to provide personal contact and health education to residents. On the other hand, vaccination providers had a closer relationship with parents in rural areas, making it convenient for them to spread vaccination information or to encourage parents to get their children vaccinated. Suitable and diverse methods of health education, or enhancements to the efficacy of general practitioner services to provide face-to-face health education might improve parent vaccination awareness and improve the inequity of vaccination coverage. 

In terms of child-specific characteristics, birth order had a negative effect on the probability of FPI. Specifically, children of a higher birth order were less likely to complete the required vaccination schedule, which was consistent with the previous reports [13,31]. This might suggest a parent’s “vaccination fatigue”, as the interest to receive childhood vaccinations waned if the number of children in the household increased. On the other hand, the results might be a reflection of the inter-sibling competition for parental care and limited household resources in a household with more children, leading to neglect of childhood vaccinations. Ethnicity was an important factor in health-seeking behaviors and outcomes. The results from the 2014 survey indicated that children of other ethnic groups were less likely to be fully immunized, while the effects appeared to have been attenuated in 2017. Our findings were in line with another study from Karachi [32]. People from other ethnic groups were considered as marginalized populations, with a lower education and socioeconomic status leading to poor awareness of primary healthcare [33]. A special focus was therefore necessary to increase vaccination awareness among the other ethnic minority groups. Immigration status made a significant contribution to the inequity of FPI coverage, with a lower coverage found in migrant children. As we know, the migrant status has been considered as a risk factor of the low vaccination coverage in Zhejiang province [34]. It was assumed that migrant parents would face challenges such as adapting to a new socio-cultural environment, living in poor economic conditions, or having a lower education level, all of which would influence the accessibility to or awareness of the utilization of the vaccination service. 

In terms of mother-specific characteristics, our results indicated an inverse relationship between the mother’s age and the probability of the FPI in 2014 and 2017, which was similar to a previous report from Africa [35]. The possible explanation was that a younger mother would have a better utilization of healthcare, including antenatal care and postnatal visits, leading to an optimal accessibility to vaccination education and a higher coverage of the primary vaccination. Employed mothers had a negative influence on the coverage of FPI, which was consistent with previous studies. Employed mothers might consider time spent waiting for vaccinations as time that could be better spent working, which reduces their propensity to visit the vaccination clinics, thereby eroding the probability of their children completing their primary immunization.

Additionally, an increased proportion of the total health spending allocated to public health spending could lead to a higher coverage of FPI. Our finding was consistent with a similar study from Taiwan [36], which indicated that the disparities in appendicitis cases narrowed due to the introduction of the national health insurance program. This evidence indicated that health service utilization, quality of service, and social inclusions would all be improved by increasing government investment in public health.

There were several limitations worth noting. First, reporting bias might occur in collecting some sensitive information, such as the monthly household income. This bias possibly underestimated the CI, since the socioeconomically advantaged people would underreport the monthly household income. Second, since we used the secondary data from two rounds of vaccination coverage surveys, the variables for the decomposition analyses were not as comprehensive as those from a special investigation. We therefore might have missed some important determinants of the inequity of FPI coverage due to this limitation. 

## 5. Conclusions

The socioeconomic inequity in the coverage of FPI still remained, although this gap was reduced between 2014 and 2017. Policy recommendations or health interventions to reduce the inequality in the coverage of FPI should be focused on eliminating poverty and women’s illiteracy, as well as controlling the other risk factors found in this study. These strategies will improve the vaccination coverage at the provincial level, and further improve the sustainable development of EPI in Zhejiang province.

## Figures and Tables

**Table 1:** Demographic and socioeconomic characteristics and the coverage of full primary immunization (FPI) of children aged 24–35 months in the 2014 and 2017 coverage surveys.

Variables	The 2014 Survey (*N* = 2772)				The 2017 Survey (*N* = 770)			
	No. of Children (%)	FPI Children			No. of Children (%)	FPI Children		
		*n* (%)	*χ* ^2^	*p*		*n* (%)	*χ* ^2^	*p*
Sex of child			0.3	0.58			0.93	0.93
Male	1395 (50.3)	1119 (80.21)			389 (50.5)	331 (85.1)		
Female	1377 (49.7)	1116 (81.05)			381 (49.5)	325 (85.3)		
Birth order of child			25.3	<0.01			12.3	<0.01
1	1952 (70.4)	1622 (83.07)			502 (65.2)	444 (88.4)		
2	651 (23.5)	490 (75.22)			229 (29.7)	182 (79.5)		
≥3	169 (6.1)	124 (73.37)			39 (5.1)	30 (76.9)		
Ethnic group of child			132.2	<0.01			49.3	<0.01
Han	2525 (91.1)	2104 (83.34)			708 (91.9)	622 (87.9)		
Others	247 (8.9)	131 (53.04)			62 (8.1)	34 (54.8)		
Age of mother (years)			0.6	0.43			10.1	<0.01
<30	1826 (65.9)	1480 (81.04)			682 (88.6)	591 (96.7)		
≥30	946 (34.1)	755 (79.81)			88 (11.4)	65 (73.9)		
Maternal education level			127.3	<0.01			19.3	<0.01
No/Primary	183 (6.6)	101 (55.08)			29 (3.8)	19 (65.5)		
Second level	1690 (61.0)	1328 (78.60)			449 (59.3)	371 (82.6)		
College level	899 (32.4)	806 (89.66)			292 (37.9)	266 (91.1)		
Maternal employment status			37.4	<0.01			9.3	<0.01
Home full-time	367 (13.2)	339 (92.38)			156 (20.3)	145 (92.9)		
Employed	2405 (86.8)	1896 (78.84)			614 (79.7)	511 (83.2)		
Residence			19.7	<0.01			8.7	<0.01
Urban	1362 (49.1)	1052 (77.24)			388 (50.4)	316 (81.4)		
Rural	1410 (50.9)	1183 (83.92)			382 (49.6)	340 (89.0)		
Immigration status			285.0	<0.01			66.9	<0.01
Resident	1661 (59.9)	1484 (89.35)			480 (62.3)	448 (93.3)		
Migrant	1111 (40.1)	751 (67.60)			290 (37.7)	208 (71.7)		
Socio-economic status			15.6	<0.01			48.2	<0.01
Q1 (poorest)	924(33.3)	713 (77.12)			257 (33.4)	191 (74.3)		
Q2 (middle)	924 (33.3)	742 (80.30)			257 (33.4)	219 (85.2)		
Q3(least poor)	924 (33.3)	780 (84.42)			256 (33.2)	246 (96.1)		

**Table 2:** The concentration index and contributions of determinants to inequities in the coverage of FPI in the 2014 coverage survey.

Variables	Level	Concentration Index (95%CI ^a^)	${m_{k}}^{b}$	${{\bar{x}}_{k}}^{c}$	% of Contribution
FPI	-	0.12028 (0.10852:0.13175)			
Birth order of child	≥3	Ref			
	2	−0.05242 (−0.06055:−0.043806)	0.11526	0.2348	−0.69
	1	0.16813 (0.15209:0.18122)	0.20509	0.7042	11.75
Ethnic group of child	Others	Ref			
	Han	0.22306 (0.21026:0.23149)	0.11283	0.9109	11.09
Maternal education level	No/primary	Ref			
	Second level	0.32109 (0.30258:0.34513)	0.50153	0.6097	29.52
	College level	0.58637 (0.55977:0.60238)	0.61027	0.3243	38.68
Maternal employment status	Home fulltime	Ref			
	Employed	−0.08262 (−0.07271:−0.088608)	0.08464	0.8676	−0.29
Residence	Urban	Ref			
	Rural	0.20651 (0.19615:0.21902)	0.18514	0.5087	9.41
Immigration status	Resident	Ref			
	Migrant	−0.26233 (−0.24583:−0.27265)	0.20227	0.4008	−10.29
GDP per-capita (CNY)	-	0.00123 (0.00110:0.00184)	0.00001	68804.68	0.41
the percentage of the total health spending allocated to public health	-	0.12255 (0.11530:0.12974)	0.01512	7.41	6.65

Note: ^a^ 95% of confidence interval; ^b^ the marginal effect of $x_{k}$; ^c^ the mean of $x_{k}$.

**Table 3:** The concentration index and contributions of determinants to inequities in the coverage of FPI in the 2017 coverage survey.

Variables	Level	Concentration Index (95%CI ^a^)	${m_{k}}^{b}$	${{\bar{x}}_{k}}^{c}$	% of Contribution
	-	0.10129 (0.095914:0.12068)			
Birth order of child	≥3	Ref			
	2	0.04031 (0.03532:0.06007)	0.05771	0.2974	1.43
	1	0.15055 (0.14211:0.17005)	0.11439	0.6519	9.28
Ethnic group of child	Others	Ref			
	Han	0.17325 (0.16452:0.18995)	0.08743		5.64
Age of mother (years)	≥30	Ref			
	<30	0.154204 (0.14398:0.16703)	0.07421	0.8857	5.09
Maternal education level	No/primary	Ref			
	Second level	0.27025 (0.26101:0.28121)	0.42557	0.5831	27.41
	College level	0.60103 (0.58912:0.61005)	0.62224	0.3792	39.72
Maternal employment status	Home fulltime	Ref			
	Employed	−0.09014 (−0.08336:−0.10065)	0.06224	0.7974	−2.73
Residence	Urban	Ref			
	Rural	0.17187 (0.16228:0.18430)	0.12426	0.4961	3.32
Immigration status	Resident	Ref			
	Migrant	−0.28303 (−0.27461:−0.29822)	0.26122	0.3766	−16.42
GDP per-capita (CNY)	-	0.00093 (0.00087:0.00119)	0.00001	72065.8	0.36
the percentage of the total health spending allocated to public health	-	0.19346 (0.18095:0.21047)	0.10133	8.32	9.27

Note: ^a^ 95% of confidence interval; ^b^ the marginal effect of $x_{k}$; ^c^ the mean of $x_{k}$.
